# Improving Parkinson’s Medicine Administration in Hospitals: The Impact of an Out-of-Hours Drug Box

**DOI:** 10.7759/cureus.48447

**Published:** 2023-11-07

**Authors:** Anum Saleem, Nutchaya Ungcharoen, Frederick Bell, Josh Storton, Hadeeqa Bibi

**Affiliations:** 1 Internal Medicine, Calderdale and Huddersfield National Health Service (NHS) Foundation Trust, Huddersfield, GBR; 2 Medical Education, Aston University, Birmingham, GBR; 3 Internal Medicine, University Hospitals Birmingham National Health Service (NHS) Foundation Trust, Birmingham, GBR; 4 Geriatrics, Calderdale and Huddersfield National Health Service (NHS) Foundation Trust, Huddersfield, GBR; 5 Pharmacy, Calderdale and Huddersfield National Health Service (NHS) Foundation Trust, Huddersfield, GBR

**Keywords:** parkinson's disease, parkinson's disease treatment, parkinson’s disease (pd), pharmacological treatment, oral levodopa

## Abstract

Background

Parkinson's disease (PD) is a progressive neurodegenerative condition characterised by the loss of dopaminergic neurons and the presence of Lewy bodies. PD medications, notably Levodopa, are critical in controlling patients' symptoms and improving their quality of life. These medications are time-critical, and a delay in administration can have a negative impact on a patient's mobility, quality of life and symptoms.

Objective

The objective of this quality improvement project (QIP) was to (1) determine the occurrence and underlying factors contributing to delays in PD medication administration at Huddersfield Royal Infirmary and (2) evaluate the impact of subsequent interventions in improving the administration of time-critical PD medications.

Methods

A total of 20 patients were recruited from Ward 5, a care of the elderly ward, at Huddersfield Royal Infirmary for the entire duration of this QIP; 10 in the pre-intervention phase and 10 for the post-intervention phase one month following the implementation of three interventions: 'Parkinson's Drug Box', ward-based teaching and a hospital-wide screensaver reminder.

Data was collected from the electronic patient record (EPR) to determine and compare the following measures before and after the interventions: the percentage of PD medications administered within the 30-minute recommended window, the admission-to-medication reconciliation time, scheduled-to-actual dose administration time, and factors causing delays.

Results

Following the implementation of three interventions, the percentage of patients receiving PD medications within the recommended 30-minute window doubled, increasing from 10% to 20%. Moreover, the average delay in patients receiving their initial PD medication dose decreased from 7.8 hours to 3.7 hours. The percentage of patients experiencing medication delays due to stock shortages also dropped from 50% to 10%.

Conclusions

Earlier medicines reconciliation and the availability of time-critical drugs out of hours are both key factors in helping to ensure PD medication is administered on time in hospitals. Staff education and a 'Parkinson's Drug Box' are two relatively simple measures that have resulted in improved outcomes for Parkinson's inpatients.

## Introduction

Parkinson's disease (PD) is the second most common progressive neurodegenerative disorder, affecting over 8.5 million individuals worldwide [1]. Over the past few years, the prevalence of PD has risen rapidly, affecting not only the patients diagnosed with the disorder but also their families, carers, and global healthcare systems [1].

The key neuropathological processes of PD are the loss of dopaminergic neurones in the substantia nigra pars compacta and the presence of Lewy bodies. These contribute to the development of both motor and non-motor symptoms, including muscle rigidity, postural instability, bradykinesia, resting tremors, and cognitive impairment. Central to PD management are dopamine replacement therapy, levodopa, and other drugs that stimulate the dopaminergic pathway, such as dopamine agonists and drugs that prevent the breakdown of dopamine, such as monoamine oxidase type B (MAO B) inhibitors and catechol-O-methyl transferase (COMT) inhibitors.

Patients with PD rely on these medications to alleviate their symptoms. As the disorder advances, a more complex and carefully timed dosing regimen becomes paramount. Annually, approximately one-third of patients with PD visit hospitals with PD-related and unrelated health issues [2]. During these visits, they often encounter delays in receiving their PD medication [3]. A study by Bakker et al. (2022) found a high rate of errors in PD medication prescription (30.5%) and administration (85%) over three years across three major South Australian hospitals. The factors contributing to these errors are multifaceted. These include incorrect prescriptions, discrepancies in the timing of drug administration, medication supply shortages, omission errors and the failure to switch to alternative non-oral forms when needed [3,4]. This switch is particularly relevant in patients who experience swallowing difficulty due to acute illnesses, are required to fast before surgery or are nil-by-mouth whilst awaiting an evaluation by the speech and language therapist (SALT) team.

The consequences of delayed medication administration, exceeding the recommended 30-minute window of the scheduled administration time [5], are significant. These include increased morbidity and mortality risks, and prolonged hospitalisation [6].

In view of these issues, this quality improvement project (QIP) was carried out at Huddersfield Royal Infirmary with two primary objectives, which were to:

(1) determine the occurrence and underlying factors contributing to delays in PD medication administration and

(2) evaluate the impact of subsequent interventions in improving the administration of time-critical PD medications. These interventions included implementing a 'Parkinson's Drug Box', ward-based education, and a hospital-wide screen saver reminder.

## Materials and methods

Approval was obtained from the Clinical Governance Department at Calderdale and Huddersfield NHS Foundation Trust prior to the commencement of the QIP.

This QIP focused on male and female patients aged 65 and above admitted to Ward 5 at Huddersfield Royal Infirmary, which had a preference for PD cases due to the Consultant Geriatrician's special interest. Ten patients admitted from 12th February 2020 were recruited, all meeting the following inclusion criteria: a diagnosis of Parkinson's disease, Lewy body dementia, or Parkinson's plus syndrome, AND regular dopaminergic medication (levodopa, dopamine agonists, MAO B inhibitors and COMT inhibitors), and direct admission from the Accident and Emergency (A&E) department to Ward 5. Patients not on at least one dopaminergic medication, who were a direct GP admission, OR transferred from another ward were excluded. Direct GP admissions were excluded as there was no clear timeline on the EPR of when they had arrived on the ward. Ward-transferred patients were also excluded, as they already had their PD medications reconciled prior to the transfer.

Data collected from Huddersfield Royal Infirmary's electronic patient record (EPR) included timing of admission, medical clerking, medication reconciliation and administration, patients' PD medication regimen, instances of missed/delayed doses with documented reasons, and recorded adverse effects. This data was reviewed to determine the following measures:

(1) Outcome measure: the percentage of PD medications administered within 30 minutes of when due.

(2) Process measures: the admission-to-medication reconciliation time, scheduled-to-actual dose administration time, and factors causing delays.

The initial data review revealed several factors contributing to PD medication administration delays: late medication reconciliation, medication shortage on the ward, and delays to the nursing drug round.

In response to this, the multidisciplinary team, comprising a Consultant Geriatrician, junior doctors, and pharmacists, planned and implemented the following three interventions:

(1) A 'Parkinson's Drug Box' containing a supply of PD medications was introduced to Ward 5 for use if there was a delay in receiving a supply from the hospital's pharmacy. This 'Box' was monitored and refilled by the Ward 5 pharmacist team.

(2) Ward-based teaching was delivered by the Consultant Geriatrician to staff, including junior doctors and nurses, during morning ward rounds.

(3) A hospital-wide screen saver with the reminder that PD medications are time critical and notice of the new Parkinson's Drug Box available in Ward 5 was introduced.

**Figure 1:**
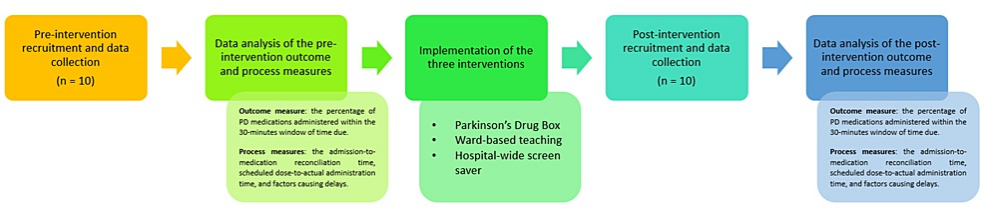
A flowchart summary of the QIP process QIP: quality improvement project, PD: Parkinson's disease

One month after the implementation of the above interventions, 10 patients in Ward 5 were recruited using the same inclusion and exclusion criteria. The same outcome and process measures were reviewed to compare if there was any improvement in the administration of time-critical PD medications (see Figure 1).

## Results

Twenty patients were recruited in the study, 10 in the pre-intervention phase and 10 in the post-intervention phase.

In the pre-intervention phase, analysis of the collected data revealed that only 10% of patients were administered PD medications within the recommended 30-minute timeframe. On average, there was a notable delay of 7.8 hours in the administration of PD medications from the scheduled time. The underlying factors contributing to the delays were identified as follows: late medication reconciliation due to prolonged waiting times for medical clerking (40%), unavailability of medication stock (50%) and delays in nursing drug rounds (10%).

Following the implementation of the interventions, several improvements were noted (see Table 1). The percentage of patients receiving PD medications within the recommended 30-minute timeframe increased to 20%. Furthermore, the average delay in the administration of the initial PD dose following admission was reduced from 7.8 hours to 3.7 hours. Notably, the delay resulting from medication stock unavailability decreased to 10%. This reduction was due to the availability of time-critical medicines from the 'Parkinson's Drug Box'. Only one instance was recorded where the box ran out of medications and was not promptly restocked. Lastly, there was a 20% reduction in documented adverse effects due to missed or delayed medications; these could range from worsening stiffness to neuroleptic malignant syndrome in severe cases.

**Table 1:** A summary comparing outcome and process measures pre- and post-intervention.

Outcome and process measures	Pre-intervention	Post-intervention
Average delay in getting initial dose	7.8 hours	3.7 hours
Average time until medicine reconciliation	12.2 hours	6.5 hours
Received dose within the recommended 30-minute timeframe	10%	20%
Delay due to unavailable medication stock	50%	10%
Missed two or more doses during their admission	40%	20%
Adverse effects from missed medications	40%	20%

## Discussion

How things should be

Timely administration of PD medication is crucial, as delays can worsen patients' motor symptoms, increase morbidity and mortality, and prolong hospital stays [6,7]. In light of this, the National Institute for Health and Care Excellence (NICE) has provided a guideline emphasising the need to administer PD medications, particularly levodopa, within 30 minutes of scheduled administration time [5].

The primary objective of this QIP was to determine whether this recommended standard is being met for inpatients at Huddersfield Royal Infirmary. Initial findings from the pre-intervention phase indicated the need for improvement; of the 10 patients randomly selected from Ward 5, only one had received their initial dose of PD medications on time. These findings were consistent with prior studies [8-14]. In an Irish study with a cohort of 46 patients in 2021, 51.7% of time-critical medications were given over 30 minutes late, and 7.5% of all doses were omitted [8]. Another study by Doyle et al. (2019) also looked at the dose administration timings and omission of PD medications within the first 48 hours of admission. They found that only nine out of the 20 patients had accurate medication reconciliation at admission, and 35% of doses were omitted or given more than 30 minutes late [9].

Barriers to timely administration of PD medications and interventions implemented

When considering the barriers to the timely administration of PD medications, it is important to evaluate the patient's journey. In an ideal scenario, patients would be triaged within 15 minutes of arrival at A&E [15]. Subsequently, an electronic alert system or triage team would then flag the patient's history of PD, facilitating early and accurate prescription of time-critical medications in the EPR. This streamlined process would ensure on-schedule PD medication.

Unfortunately, the results of the pre-intervention phase did not reflect this, with 40% of the patients receiving their PD medication late due to a delay in prescribing. The reasons for this delay are multifactorial, with one key reason being prolonged waiting times in A&E before patients are clerked and prescribed the necessary medications. A recent article by the King's Fund highlighted the severity of this issue, noting that only half of the patients attending A&E in the UK were seen within four hours [16]. The causes behind these long waiting times include staff shortage, high hospital bed occupancy and a surge in emergency attendance [16]. In addition to prolonged waiting time, there may be circumstances where patients are acutely unwell, or experience altered consciousness and cannot self-administer their PD medications while waiting to be seen. Furthermore, when reviewed, they may not have access to their medication supply and/or are unable to recall their drug history. These factors, as identified in our QIP and other studies, contribute to PD medication delays [3,4,8].

Furthermore, a notable observation from this QIP was that doctors in the A&E department often defer medication reconciliation to the clerking medical team. Instead, they opted to prescribe medications on a 'once-only' basis for the patient's duration in A&E. This approach has become increasingly common due to the time pressure caused by a rapid rise in the number of emergency attendances post-COVID-19 pandemic. While this prescribing approach might not seem problematic when medical clerking is promptly undertaken, the reality is that many patients spend several hours waiting to be seen, referred, and admitted by an appropriate team [16]. Our pre-intervention findings echoed this issue, revealing that the average duration between admission and medical clerking and medication reconciliation at Huddersfield Royal Infirmary was nine hours.

Another key issue identified in the pre-intervention phase was that 50% of patients experienced delayed PD medication due to a supply shortage. This resulted from the inpatient hospital pharmacy closing at 5 p.m., resulting in nursing staff relying on limited ward stock after hours. A key intervention in this QIP has been the implementation of the 'Parkinson's Drugs Box' in Ward 5, which contained a readily available supply of PD medication to be used as needed. The impact of this was considerable, with post-intervention data showing the percentage of patients experiencing medication delays due to stock unavailability reduced from 50% to 10%.

In addition to implementing the 'Parkinson's Drug Box', ward-based and staff-wide education on the need for timely administration of PD medication was introduced and led by the Ward 5 multidisciplinary team. These educational interventions were delivered as a hospital-wide screensaver reminder and consultant-led ward round teaching. Collectively, these three intervention approaches had a positive impact on the timely PD medication administration.

Limitations and areas of future projects

This QIP has been designed to provide a 'snapshot' of the inpatient care received by patients with PD in Ward 5 at Huddersfield Royal Infirmary. While the small sample size of 10 patients for both the pre- and post-intervention phases has advantages in data collection efficiency, we acknowledge that this may compromise the validity and reliability of the results, as larger studies tend to have lower standard deviations.

Going forward, conducting another 'snapshot' QIP using the same methodology but with a larger sample size would be useful. This is to find out whether the interventions have resulted in a lasting change in staff culture and patient outcomes or if this was merely a temporary improvement. Potential interventions that may be of benefit and warrant further QIPs to be conducted include:

• Establishing a hospital-wide system to alert Parkinson's specialist clinicians and nurses upon patient admission.

• Extending the availability of the 'Parkinson's Drug Box' to the A&E department.

• Empowering patients to self-administer PD medications when possible while waiting for a medical clerking.

These intervention measures align with the recommendations outlined in the 'Get it on time' report [4].

## Conclusions

Delays in the administration of PD medications often occur due to late medication reconciliation and shortage of medication supply.

Introducing a 'Parkinson's Drug Box' has positively affected the administration of time-critical medications, resulting in better patient outcomes and reduced adverse effects.

Going forward, a system must be in place to ensure the 'Parkinson's Drug Box' remains stocked and reinforce the '30-minute rule' with newly rotating clinical team members.

Further 'snapshot' audits should be conducted to monitor the long-term outcomes of the interventions.
